# FOXM1 regulates expression of eukaryotic elongation factor 2 kinase and promotes proliferation, invasion and tumorgenesis of human triple negative breast cancer cells

**DOI:** 10.18632/oncotarget.7672

**Published:** 2016-02-24

**Authors:** Zuhal Hamurcu, Ahmed Ashour, Nermin Kahraman, Bulent Ozpolat

**Affiliations:** ^1^ Department of Experimental Therapeutics, The University of Texas MD Anderson Cancer Center, Houston, Texas, USA; ^2^ Faculty of Medicine, Department of Medical Biology, Erciyes University, Kayseri, Turkey; ^3^ Betül-Ziya Eren Genome and Stem Cell Center, Erciyes University, Kayseri, Turkey; ^4^ Center for RNA Interference and Non-Coding RNA, The University of Texas MD Anderson Cancer Center, Houston, Texas, USA

**Keywords:** breast cancer, triple-negative breast cancer, TNBC, FOXM1, eEF2-kinase

## Abstract

Eukaryotic elongation factor 2 kinase (eEF2K), an emerging molecular target for cancer therapy, contributes to cancer proliferation, cell survival, tumorigenesis, and invasion, disease progression and drug resistance. Although eEF2K is highly up-regulated in various cancers, the mechanism of gene regulation has not been elucidated. In this study, we examined the role of Forkhead Box M1 (FOXM1) proto-oncogenic transcription factor in triple negative breast cancer (TNBC) cells and the regulation of eEF2K. We found that FOXM1 is highly upregulated in TNBC and its knockdown by RNA interference (siRNA) significantly inhibited eEF2K expression and suppressed cell proliferation, colony formation, migration, invasion and induced apoptotic cell death, recapitulating the effects of eEF2K inhibition. Knockdown of FOXM1 inhibited regulators of cell cycle, migration/invasion and survival, including cyclin D1, Src and MAPK-ERK signaling pathways, respectively. We also demonstrated that FOXM1 (1B and 1C isoforms) directly binds to and transcriptionally regulates eEF2K gene expression by chromatin immunoprecipitation (ChIP) and luciferase gene reporter assays. Furthermore, *in vivo* inhibition of FOXM1 by liposomal siRNA-nanoparticles suppressed growth of MDA-MB-231 TNBC tumor xenografts in orthotopic models. In conclusion, our study provides the first evidence about the transcriptional regulation of eEF2K in TNBC and the role of FOXM1 in mediating breast cancer cell proliferation, survival, migration/invasion, progression and tumorgenesis and highlighting the potential of FOXM1/eEF2K axis as a molecular target in breast and other cancers.

## INTRODUCTION

Breast cancer is the most common malignancy and the second leading cause of cancer- related death in women [1]. Patient deaths are attributed to lack of therapeutic options following development of resistance to standard therapies (i.e., chemotherapy and radiotherapy) and metastatic disease [2]. The poor outcomes, especially in triple-negative breast cancer (TNBC), are largely due to lack of known therapeutic targets (e.g., estrogen receptor [ER] or human epidermal growth factor receptor 2 [HER2]), highlighting the crucial need to better understand the biology of this complex cancer and to develop better therapeutic strategies to improve patient survival [2].

Forkhead Box M1 (FOXM1), a member of the FOX protein family characterized by a conserved winged-helix DNA binding domain [3], promotes cell cycle progression by inducing both transition from G1 to S phase and transition from G2 to M phase [4–11]. Recent studies demonstrated that elevated FOXM1 expression is found in a wide variety of cancers, including breast, ovarian, colon, liver, pancreatic and gastric cancers; Ewing sarcoma; hepatocellular carcinoma, and cervical cancer [10–16]. Moreover, expression of FOXM1 is correlated with a clinically aggressive, drug-resistant, cancer phenotype and poor patient survival [17–21]. FOXM1 is regulated by oncogenic signals, including growth factors, and suppressed by p53 tumor suppressor protein, which is mutated in half of human cancers [22, 23]. FOXM1 is considered an emerging target in breast cancer due to its oncogenic role and high overexpression rate in 85% of TNBCs, which also have a *high* p53 mutation frequency (~80% of cases) [24–31]. Recently, analysis of The Cancer Genome Atlas (TCGA) breast cancer database also identified FOXM1 as the key transcriptional driver in the differentially expressed gene signature of TNBC [29, 31], demonstrating the significance of FOXM1 as a driver of proliferation and disease progression [29].

Eukaryotic Elongation Factor 2 Kinase (eEF2K), a Ca^2+^/calmodulin-dependent Ser/Thr kinase that regulates protein synthesis through phosphorylation of eEF2 [32–35], has been shown to be involved in mediating autophagy and cell survival in nutrient deprivation and hypoxia [32, 33]. Recently, we showed that eEF2K promotes TNBC cell proliferation, invasion and tumorigenesis, and resistance to chemotherapy by inducing oncogenic signaling pathways related to cell growth, survival, invasion, angiogenesis, and epithelial-mesenchymal transition in pancreatic cancer [36–38]. eEF2K is overexpressed in solid cancers, including pancreatic and colon cancer and glioblastoma, and correlates with poor patient survival [36–40]. Therapeutic inhibition of eEF2K prevents tumor growth and enhances the efficacy of chemotherapy in pre-clinical TNBC models in mice [36], indicating that eEF2K is an important regulator of tumor growth and progression and a potential therapeutic target in TNBC. However, the molecular mechanism regulating eEF2K gene expression remains largely unknown.

In this study we investigated the role of FOXM1 oncogenic transcription factor in TNBC biology and regulation of eEF2K. We demonstrated that FOXM1 transcriptionally regulates eEF2K by binding to the promoter region and mediates some of the tumorigenic effects of FOXM1, as down-regulation of FOXM1 inhibited cell proliferation, colony formation, migration/invasion and tumorigenesis, recapitulating the effects of eEF2K down-modulation in TNBC.

## RESULTS

### FOXM1 expression is highly up-regulated in breast cancer cells

**Figure 1:**
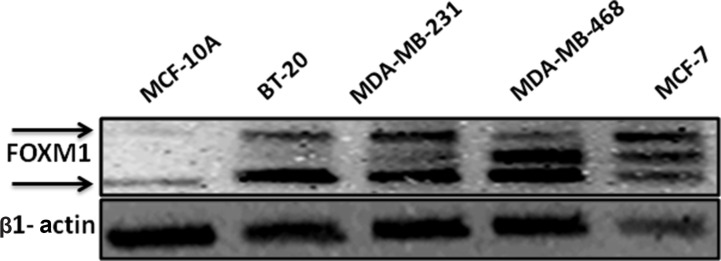
FOXM1 protein is overexpressed in TNBC cells Breast cell lines were analyzed by Western blot using a specific antibody against FOXM1. β-Actin was used as a loading control. FOXM1 protein was highly expressed in breast cancer cell lines compared with its expression in non-tumorigenic human breast cells (MCF10A).

The baseline expression of FOXM1 was determined in various human breast cancer cell lines and non-tumorigenic human breast cells (MCF10A) by Western blot analysis. Triple negative breast cancer (TNBC) cells (MDA-MB-231, BT-20) had higher FOXM1 expression than other breast cancer cell lines, including the ER-positive T-47D, and ZR-75.1 cells and the HER2/Neu-positive SKBR3 cells (Figure 1 and Supplementary Figure 1). BT-20 and MDA-MB-231 cells were therefore used for all subsequent experiments.

### Inhibition of FOXM1B and FOXM1C isoforms suppresses cell growth and colony formation in TNBC cells

Transcription of the FOXM1 locus results in three differentially spliced mRNAs, leading to expression of three isoforms of FOXM1 (1-A, 1-B, and 1-C). Although all three isoforms of FOXM1 can bind to DNA, only FOXM1-B and FOXM1-C have been shown to be transcriptionally active (5, 42). FOXM1-A, which contains extra A1 and A2 domains from exons Va and exon VIIa, respectively, is transcriptionally inactive due to the presence of an A2 domain that disrupts the transactivation activity [43].

**Figure 2:**
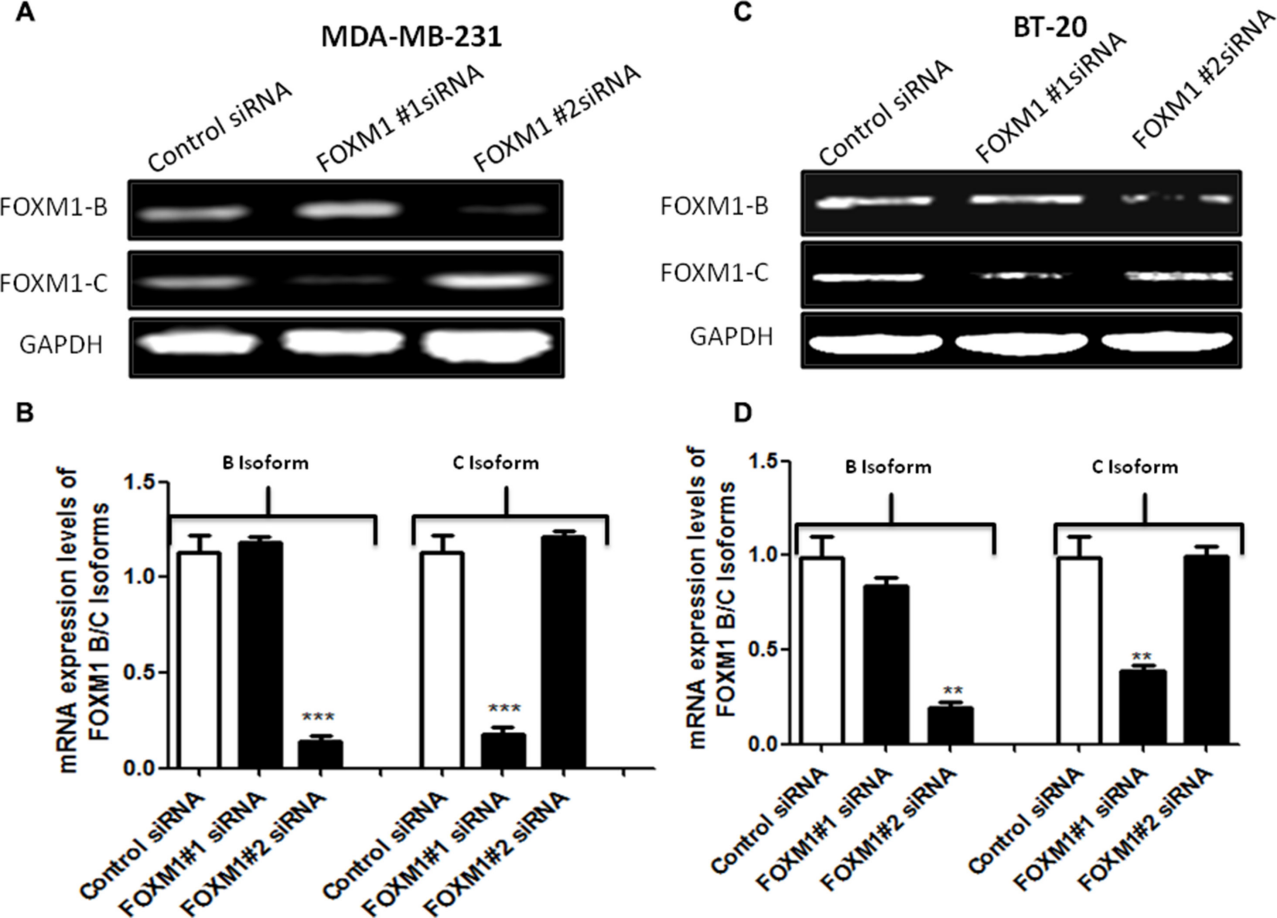
siRNA specifically targeting FOXM1 inhibits FOXM1 expression in both MDA-MB-231 and BT-20 cells Cells were transfected with 50 nM FOXM1#1 or FOXM1#2 siRNA or control siRNAs. Total RNA was isolated 72 h after transfection, and FOXM1 mRNA levels were determined by RT-PCR. The two different FOXM1 siRNAs significantly inhibited the expression of FOXM1 mRNA in both MDA-MB-231 (**A**, **B**) and BT-20 (**C**, **D**) cells. FOXM1#1 siRNA inhibited FOXM1-C, and FOXM1#2 siRNA suppressed FOXM1-B. GAPDH expression was used as a control. Data are represented as mean ± SD. *represents significant difference between indicated groups

The cells were transfected with control siRNA or two different FOXM1 siRNAs, and total RNA was isolated 72 h after the transfection and analyzed by RT-PCR. Compared with the expression of FOXM1-B and -1-C in control siRNA-transfected cells, the expression of the two isoforms was suppressed in cells transfected with two different FOXM1 siRNA (Figure 2). These results showed that the FOXM1 siRNA can effectively knock down FOXM1 expression at the transcriptional level and be used to study FOXM1-mediated effects.

**Figure 3:**
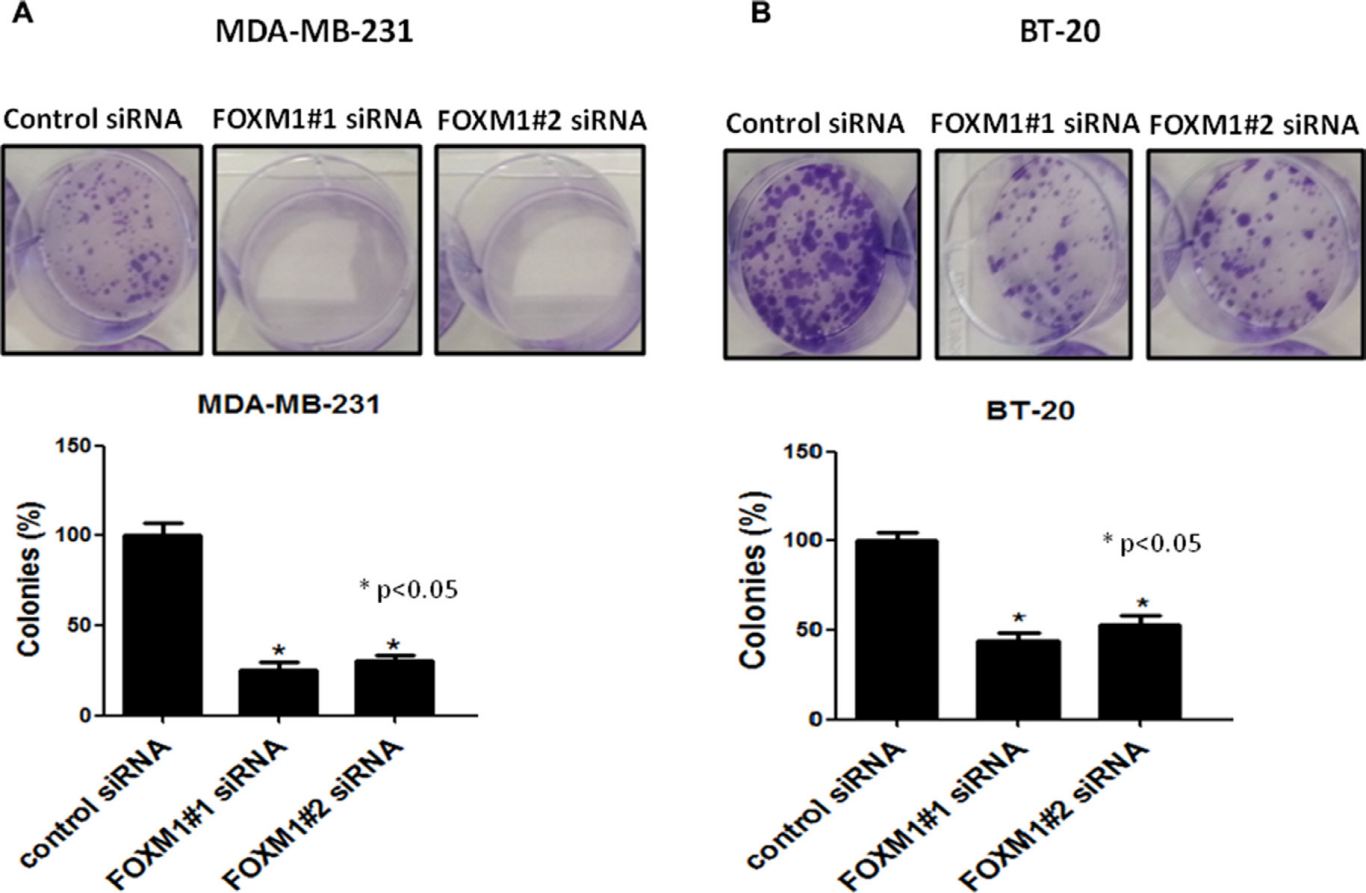
Effect of FOXM1 knockdown on colony formation Knockdown of FOXM1 by siRNA (50 nM) significantly inhibited colony formation in both MDA-MB-231 (**A**) and BT-20 (**B**) cells. Cell colonies were stained with crystal violet and the colonies-area distribution regions were measured densitometrically at the end of the two weeks. The histograms show the percentages of the formed colonies. Data is expressed as mean of percentages of colony formation ± SD of three independent experiments. *represents significant difference between indicated groups.

We next examined the effects of FOXM1-B (FOXM1#2) and FOXM1-C (FOXM1#1) siRNAs on cell proliferation and colony formation in MDA-MB-231 and BT-20 cells by using a clonogenic assay, which measures the ability of tumor cells to grow and form foci [44]. In this assay, normal cells are contact inhibited and do not form colonies, while cancer cells do form colonies; thus, the assay measures the neoplastic propensity of cancer cells. Clonogenicity was evaluated by plating fixed numbers of cells into tissue culture dishes and culturing for 2 weeks. Down-regulation of FOXM1B and -1C by specific siRNAs resulted in a marked reduction of colony formation compared with colony formation in the control siRNA-transfected MDA-MB-231 and BT-20 cells (*p* < 0.05, Figure 3). We also examined the short-term effects of FOXM1-B and FOXM1-C siRNAs on proliferation of the two cell lines at 72 h by MTS assay. Down-regulation of FOXM1 significantly reduced the proliferation of MDA-MB-231 cells compared with the control siRNA-transfected cells (*p* < 0.001, Supplementary Figure 2). These results demonstrated that expression of the FOXM1 transcription factor is essential for proliferation and colony formation of TNBC cells.

### Knockdown of FOXM1 impairs cell motility, migration and invasion of TNBC cells

**Figure 4:**
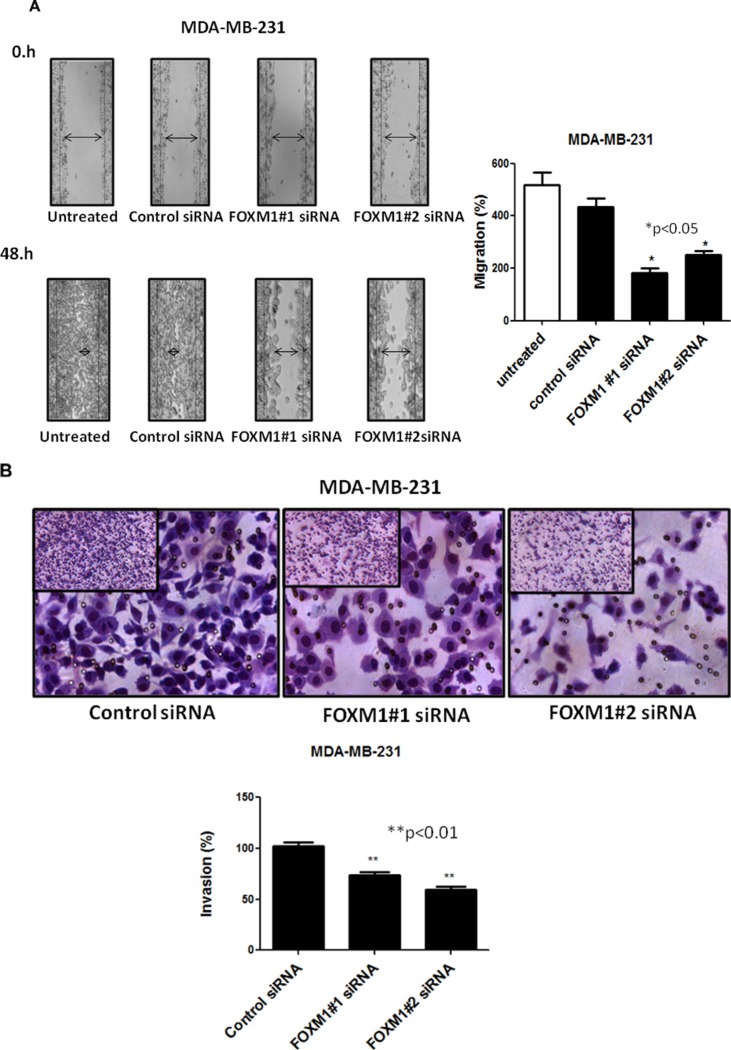
Effect of down-regulation of FOXM1 on invasion and migration of MDA-MB-231 cells Cell migration was measured by a scratch wound healing assay. A single scratch was made in the center of the confluent cell monolayer, and the wounded monolayers were transfected with indicated siRNAs. The wounds repair was monitored for 12 and 48 h and visualized microscopically with original magnification 6100. Images were taken immediately (0 h), and after 48 h of scratching the cultures. The histogram shows the percentages of the cells migration, and the data is expressed as mean of the percentages of migration ± SD of three independent experiments (**A**). MDA-MB-231 cells were transfected with 50 nM of indicated siRNAs (for 72 h), and equal numbers of viable cells were seeded onto Matrigel-coated Transwell filters in Matrigel invasion chambers. The number of the cells that invaded after 24 h was determined as in protocol. Magnification,1006. The histograms show the mean of percentages of invasion ± SD of three experiments. *represents significant difference between indicated groups.

To assess whether FOXM1 is involved in cell motility and migration of TNBC cells, we performed an *in vitro* scratch wound healing assay. MDA-MB-231 cells were plated in a six-well plates and transfected with siRNA targeting FOXM1-B or -C; 72 h after transfection, a single scratch wound was created in the well, and the cells were monitored for 48 h. Cells treated with control siRNA were able to migrate and completely close the wound, while cells treated with FOXM1 siRNA had limited migration and did not fill the gap after 48 h (Figure 4A, *p* < 0.05). Next, we examined if FOXM1 is involved in cell invasion by performing *in vitro* cell invasion assays using Matrigel-coated Boyden chambers. We found that down-regulation of FOXM1 by the two FOXM1 siRNAs inhibited the invasiveness of MDA-MB-231cells compared to the invasiveness of the control siRNA-transfected cells, with markedly fewer cells invading the bottom well (*p* < 0.01, Figure 4B), indicating the involvement of FOXM1 in cell invasiveness. Overall, our findings suggest that FOXM1 promotes cell motility, migration, and invasion in TNBC cells.

### Down-regulation of FOXM1 promotes apoptotic death of TNBC cells

**Figure 5:**
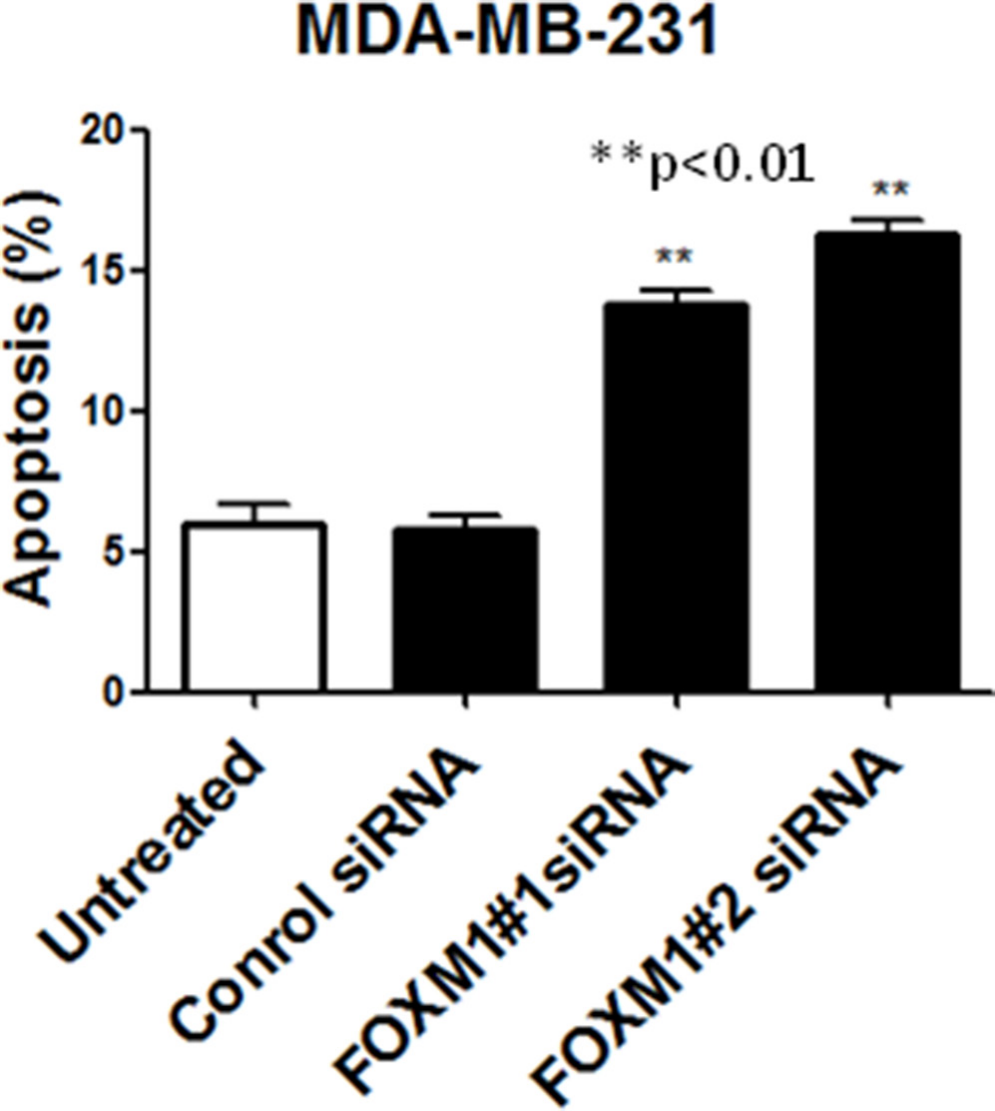
FOXM1 down-regulation induces apoptosis in TNBC cells MDA-MB-231 cells were transfected with FOXM1#1 or FOXM1#2 siRNA (50 nM) and analyzed by annexin V/propidium iodide staining, and positively stained cells were quantified by FACS. The histogram show percentage of apoptotic cells in relation to the total number of cells. *represents significant difference between indicated groups.

To determine whether FOXM1 is involved in cell survival and whether inhibition of FOXM1 induces apoptosis, we knocked down FOXM1 in MDA-MB-231 cells, using the two different FOXM1 siRNAs and assessed apoptotic cell death. Apoptosis was quantitatively analyzed using annexin V/propidium iodide staining assay. Down-regulation of FOXM1 resulted in a significant increase in the number of apoptotic MDA-MB-231 cells (*p* < 0.01, Figure 5), suggesting that expression of FOXM1 promotes cell survival and prevents apoptotic cell death in breast cancer cells.

### FOXM1 regulates eEF2K expression in TNBC cells

We recently demonstrated that eEF2K promotes breast cancer cell proliferation, clonogenicity, invasion, tumorigenesis, and resistance to chemotherapeutics by inducing clinically significant signaling pathways [45]. Because FOXM1 is also involved in these functions in breast cancer cells, we hypothesized that FOXM1 may transcriptionally regulate eEF2K expression.

**Figure 6:**
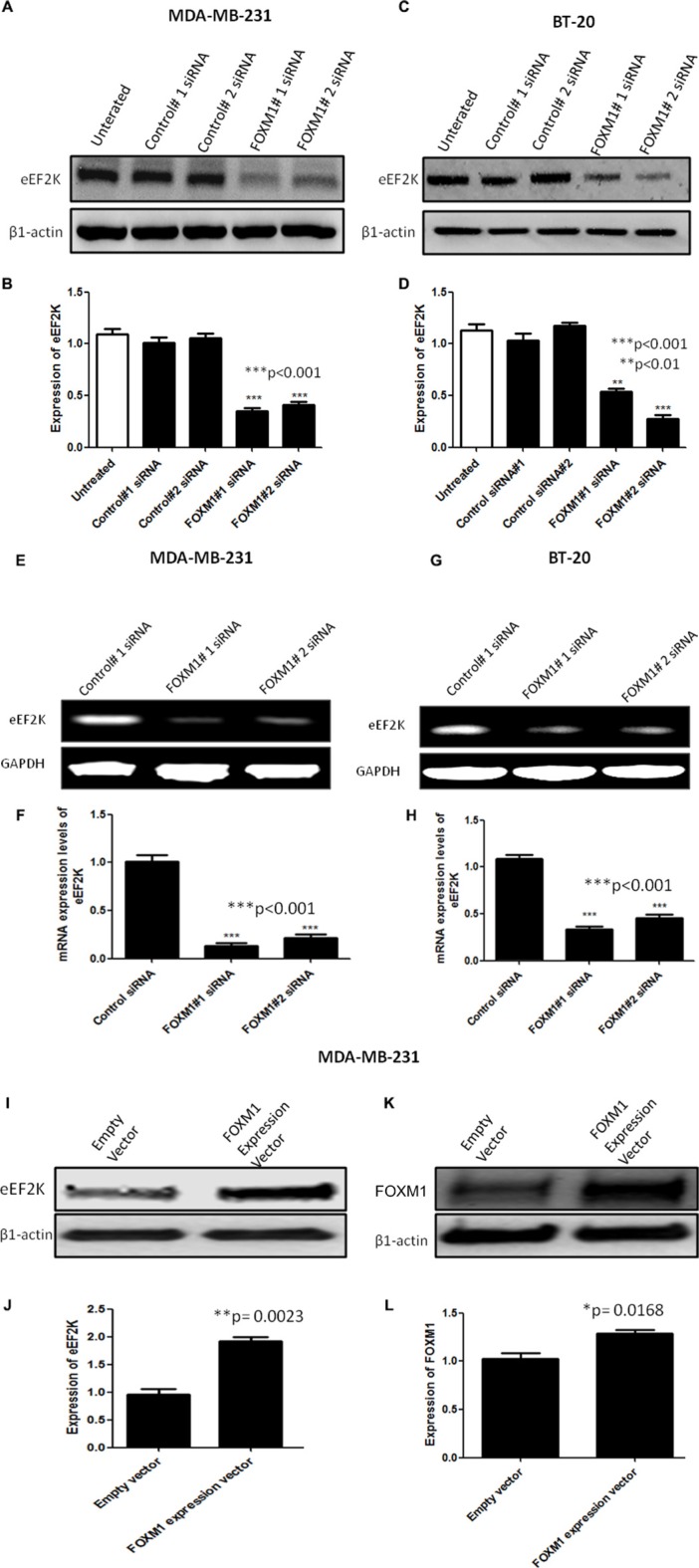
FOXM1 regulates EF2K mRNA and protein expression Cells were transfected with 50 nM FOXM1#1 or FOXM1#2 siRNA or control siRNA. Protein extracts were isolated 72 h after transfection. eEF2K protein levels were determined by Western blot analysis using anti-eEF2K monoclonal antibody. β1-Actin was used as a loading control. siRNA-mediated silencing of FOXM1 inhibited eEF2K expression in both MDA-MB-231 (**A**, **B**) and BT-20 (**C**, **D**) cells. Total RNA was isolated 72 h after transfection. eEF2K mRNA levels were determined by standard RT-PCR. Band intensities indicated obvious down-regulation of eEF2K transcription in both MDA-MB-231 (**E**, **F**) and BT-20 (**G**, **H**) cells. GAPDH was used as a loading control. In addition, MDA-MB-231 cells were transfected with a FOXM1 expression vector (400 ng) or an empty vector. eEF2K (**I**, **J**) and FOXM1 (**K**, **L**) protein expression levels were determined by Western blot analysis. β1-Actin was used as a loading control. The data are means with standard deviations. *represents significant difference between indicated groups.

To test this hypothesis, we conducted Western blot and RT-PCR analyses to determine if the expression levels of eEF2K protein and mRNA, respectively, are inhibited after FOXM1 is silenced by siRNA. Knockdown with FOXM1-B or -1-C siRNA significantly inhibited expression of both eEF2K protein and eEF2K mRNA in both MDA-MB-231 and BT-20 cells (Figure 6A–6H).

To determine the effect of increased FOXM1 expression on eEF2K expression, we transfected MDA-MB-231 cells with the FOXM1 expression vector or the empty vector. We found that these cells exhibited significantly increased eEF2K protein expression (Figure 6I–6L). Overall, these results suggest that FOXM1 is involved in eEF2K expression in breast cancer cells.

### FOXM1 binds to the eEF2K promoter and transcriptionally regulates its expression

**Figure 7:**
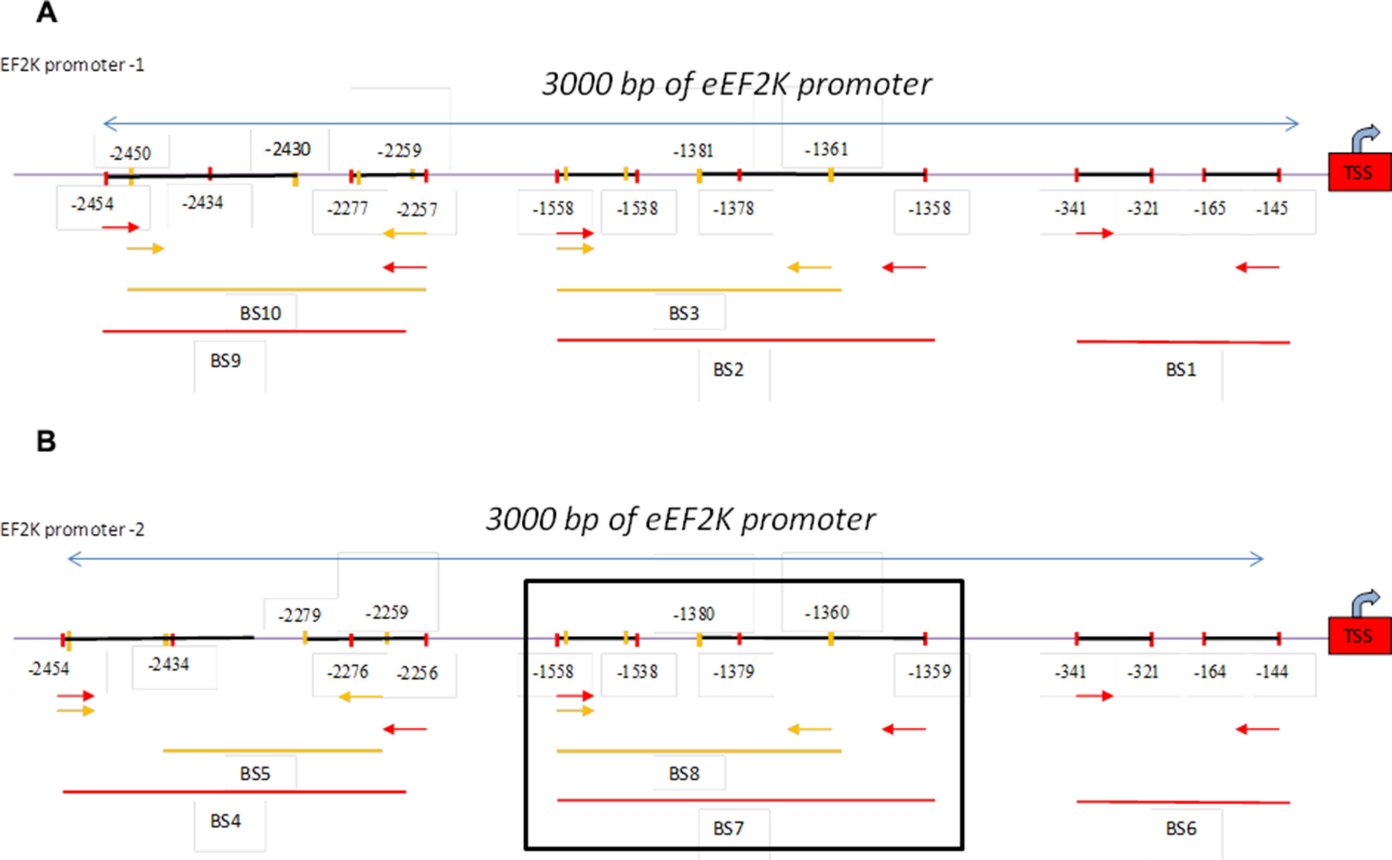
Schematic representation of the human eEF2K gene promoter Two EF2K promoter regions were determined (used http://genome.ucsc.edu). One of them, promoter-1, the other promoter was indicated as promoter-2. Ten different primers were designed to two promoter region of EF2K gene using Primer 3 program. The 3000-bp sequence is numbered from the transcription start site (TSS) (+1). Primers BS1 to -10 are indicated. Primers BS1, BS2, BS3, BS9, BS10 were designed for promoter-1 region (Figure 7A) and primers BS4, BS5, BS6, BS7, BS8 were designed for promoter-2 (Figure 7B).

**Figure 8:**
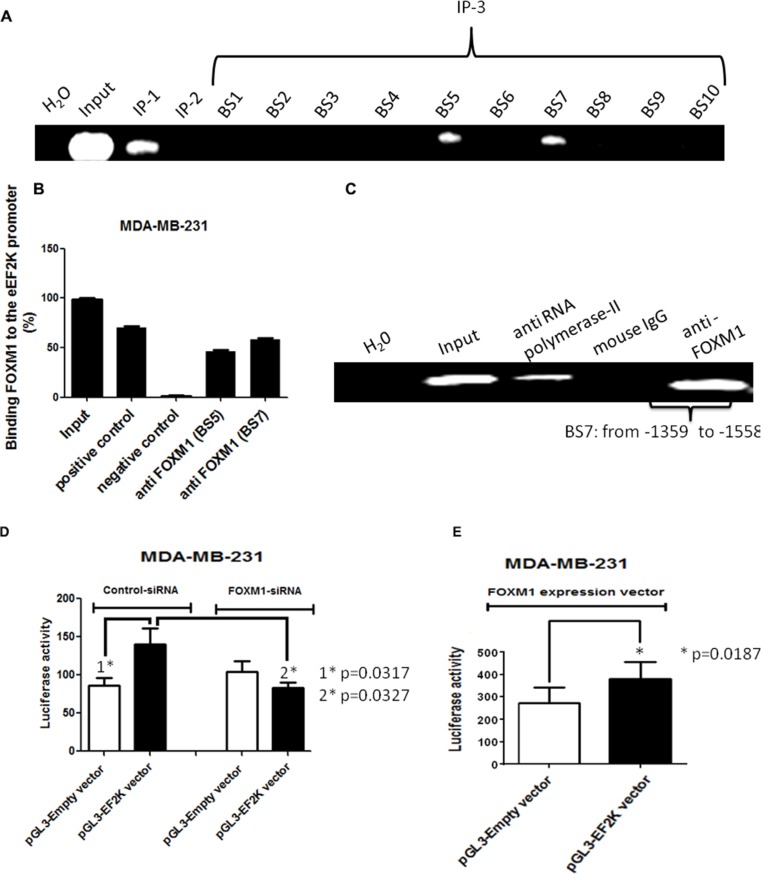
The human eEF2K gene promoter is a direct transcriptional target of FOXM1 Protein-DNA complexes from MDA-MB-231 cells were cross-linked by using formaldehyde and sonicated. (**A**, **B**) Chromatin fragments from these cells were immunoprecipitated (with antibodies specific to RNA polymerase II (immunoprecipitation 1 [IP-1]; positive control), mouse IgG (IP-2; negative control), and FOXM1 (IP-3) as indicated. Input, total DNA. After reversal of cross-linking, the immunoprecipitated DNA was amplified by PCR using the specific primers and resolved on 1.2% agarose gels. ChIP assay demonstrated that FOXM1 protein bound to the promoter region of the eEF2K gene. (**C**) To confirm this finding, immunoprecipitated DNA was analyzed by PCR with primers specific to eEF2K gene promoter fragment containing BS7. The assay results confirmed the binding of FOXM1 to the eEF2K gene promoter. (**D**, **E**) Cells were transfected with 50 nM FOXM1 siRNA or control siRNA (D) or with a FOXM1 expression vector and pRTLK vector (renilla) (E) and either the pGL3 empty vector or the pGL3-EF2K vector. Cells were harvested 40 h after transfection, and protein extracts were preapared and analyzed for dual-luciferase activity. Triplicate plates were used to calculate the mean fold induction of transcriptional activity. The luciferase activity values are relative to the activity of the cotransfected renilla luciferase. The luciferase reporter assay demonstrated that inhibition of FOXM1 expression led to down-regulation of eEF2K activity and that expression of FOXM1 resulted in increased eEF2K activity. The data are means with standard deviations. *represents significant difference between indicated groups.

To provide a direct link between FOXM1 and eEF2K and determine whether FOXM1 transcriptionally regulates eEF2K expression, we investigated if FOXM1 binds to the promoter region of the eEF2K gene. To this end, we performed standard ChIP assays. Briefly, using the Align Sequences Nucleotide BLAST program, we looked for the consensus FOXM1-binding sequence in the promoter sequence of the eEF2K gene. We found four different FOXM1-binding consensus sequences in the eEF2K promoter region (Supplementary Figure 3). Next, the cross-linked and sonicated human chromatin prepared from MDA-MB-231 cells was immunoprecipitated with antibodies specific to either FOXM1 or RNA polymerase. Normal mouse IgG serum was used as a negative control. The genomic DNA associated with the immunoprecipitated chromatin was amplified by PCR with primers specific to the human eEF2K gene promoter region, as described in Materials and Methods (Figure 7). As shown in Figure 8A–8B, anti-FOXM1 antibody, but not the control antibodies, precipitated the EF2K promoter fragment containing the BS7 sequence (−1359 to −1558; Figure 7B). We also performed a control ChIP assay. Specific primers were designed to amplify the promoter fragment containing the BS7 sequence, and the immunoprecipitated DNA was analyzed by PCR. The ChIP experiment demonstrated that FOXM1 bound to eEF2K promoter (Figure 8C).

To further investigate whether FOXM1 regulates eEF2K promoter activity, we used the luciferase reporter assay. We knocked down FOXM1 by co-transfecting FOXM1 siRNA and the plasmid vector pGL3 incorporating eEF2K promoter into MDA-MB-231 cells. Conversely, to assess the effect of increased FOXM1 expression on eEF2K transcription, we also co-transfected cells with the FOXM1 expression vector and the pGL3-eEF2K promoter vector. We prepared protein extracts from MDA-MB-231 cells at 40 h following transfection and measured dual-luciferase activity. The co-transfection experiments with FOXM1 siRNA demonstrated that eEF2K promoter activity was reduced, while FOXM1 expression led to an increase in eEF2K promoter activity (Figure 8D–8E). Taken together, the results from the ChIP and luciferase reporter assays demonstrated that the eEF2K promoter region is a direct transcriptional target of FOXM1 and that FOXM binds to the promoter of the eEF2K gene and induces eEF2K expression.

### Knockdown of FOXM1 inhibits mediators of the cell cycle, cell survival, and cell invasiveness in TNBC cells

**Figure 9:**
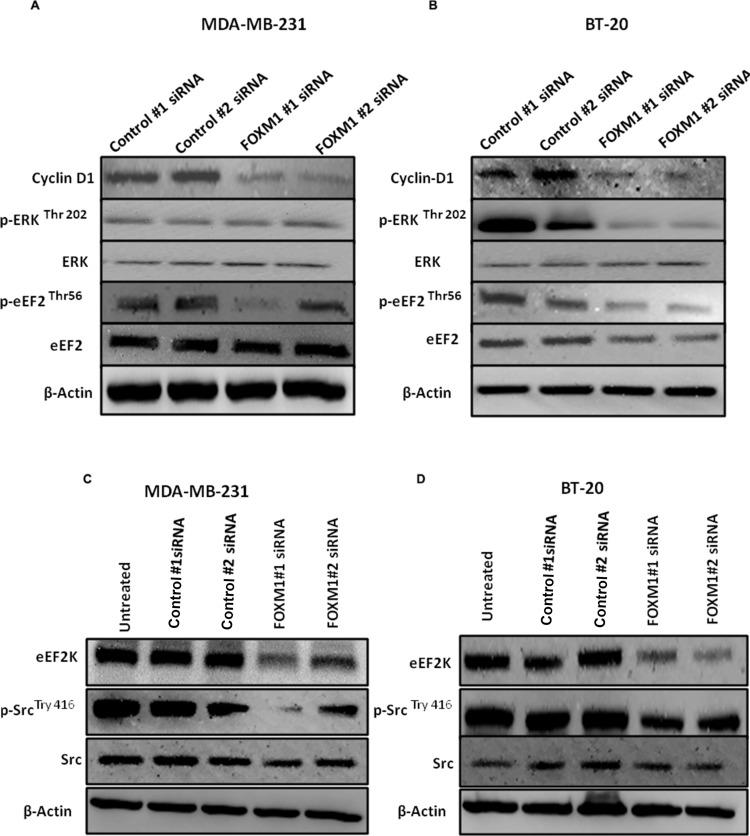
Down-regulation of FOXM1 inhibits expression of downstream molecular targets of eEF2K Cells were transfected with 50 nM FOXM1#1 or FOXM1#2 siRNA or control siRNA. Protein extracts were isolated 72 h after transfection. β1-Actin was used as a loading control. (**A**, **B**) Knockdown of FOXM1 by siRNA decreased expression levels of Cyclin D in both cell lines. FOXM1 knockdown additionally by siRNA reduced expression levels of p-ERK (Thr-202) in BT-20 cells but not in MDA-MB-231 cells. The expression level of ERK was not changed in either MDA-MB-231 or BT-20 cells. Knockdown of FOXM1 also inhibited p-eEF2 levels (Thr-56), but the expression level of eEF2 was not changed in either MDA-MB-231 or BT-20 cells. (**C**, **D**) Knockdown of FOXM1 suppressed the protein levels of eEF2K and p-Src (Tyr-416) but not Src in both cell lines.

As indicated by the data in Figure 3 and Supplementary Figure 2, FOXM1 plays a critical role in cell proliferation and cell cycle progression in TNBC cells. We also examined whether inhibition of FOXM1 leads to inhibition of signaling pathways or mediators of these cellular events. We first examined previously reported indirect downstream targets of eEF2K [36] in breast cancer cells. Transfection with FOXM1#1 or #2 siRNA reduced the levels of cyclin D1 (which promotes the cell cycle entry by inducing G1/S phase transition), p-Src-Tyr416 (which is one of the most important mediators of cell invasion), and pERK-EF2-Thr56 (a direct target phosphorylated by eEF2K), which as components of the ERK-MAPK pathway, control cell proliferation [46] (Figure 9). Western blot analysis revealed that knockdown of FOXM1 markedly reduced p-ERK in BT-20 cells but not in MDA-MB-231 cells (Figure 9A–9B), indicating that FOXM1 regulates eEF2K and its downstream molecular targets in TNBC.

### *In vivo* therapeutic targeting of FOXM1 by siRNA inhibits growth of orthotopic TNBC xenograft tumors in mice

**Figure 10:**
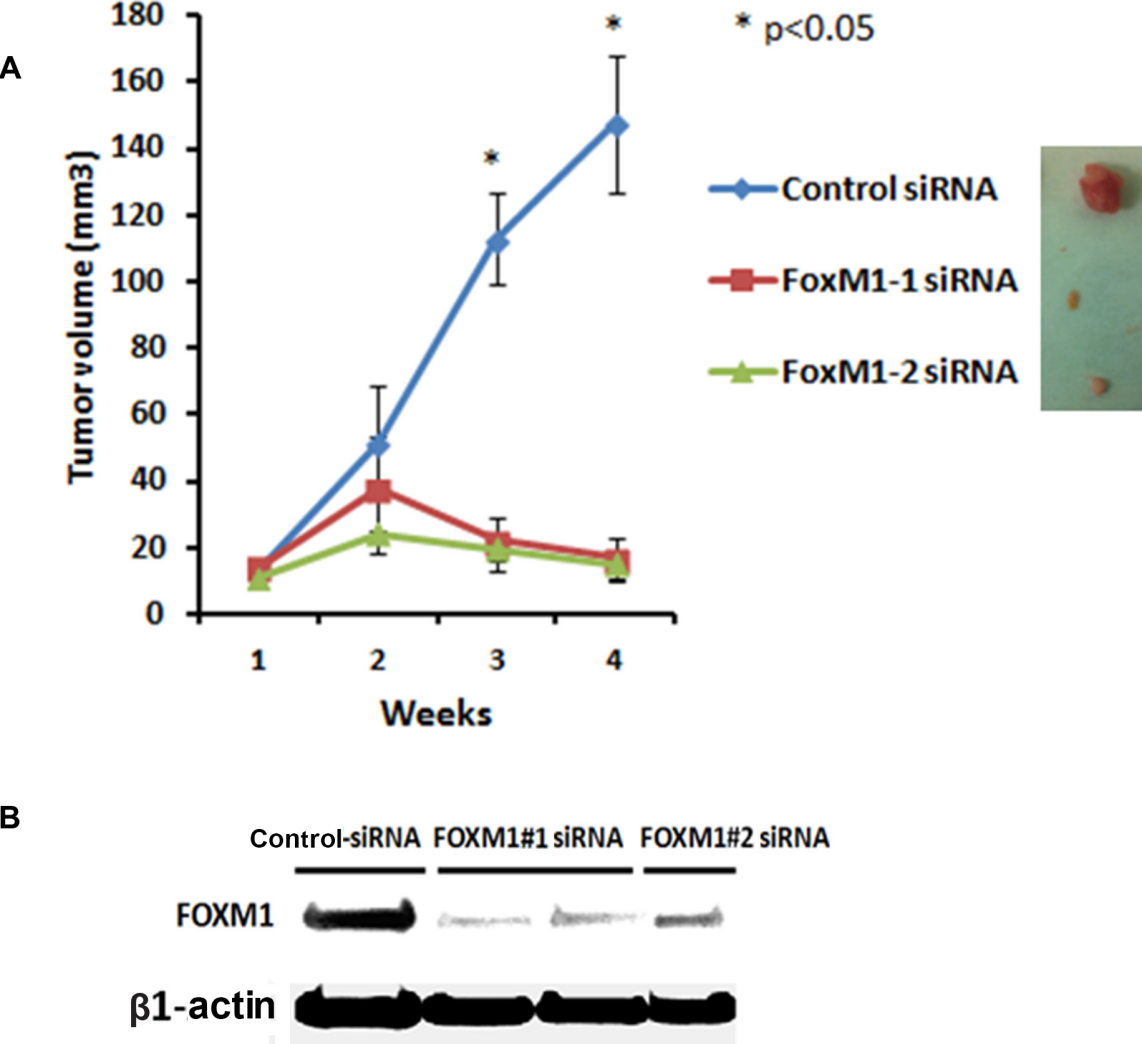
Systemic administration of liposomal FOXM1 siRNA inhibits tumor growth in an orthotopic mouse MDA-MB-231 xenograft model (**A**) MDA-MB-231 cells were injected into mammary fat pat of female athymic nude mice. Mice were either treated with liposomal nanoparticles incorporating control siRNA or FOXM siRNA (#1, #2) (0.3 mg/kg or about 8 μg/mouse, i.v once a week from tail vein, *n* = 5 mice/group). Tumors sizes were measured weekly. The tumor volumes growth curve of MDA-MB-231 are shown as mean ± SD of the group and ±SEM, *p*-values obtained with Student's *t*-test. (**B**) Tumor samples of MDA-MB-231 xenografts from control siRNA and FOXM1siRNA -treated mice were analyzed by Western blot analysis for FOXM1 expression.

To determine the *in vivo* role of FOXM1 in the TNBC tumorigenesis and its potential as a therapeutic target we further assessed the effects of FOXM1 inhibition in an orthotopic xenograft model MDA-MB-231 in mice. MDA-MB-231 cells were implanted into the mammary fat pad of nude mice by subcutaneous injection and about 2 weeks later two different FOXM1 siRNA (#1 and #2) and non-silencing control siRNA (0.3 mg/kg, i.v. from tail vein) incorporated in DMPC-based nanoliposomes were injected into mice once a week for four weeks. As shown in Figure 10, mice treated with liposomal FOXM1 siRNA had significantly smaller tumors compared with those treated control siRNA treated group (*n* = 5 animals/group, *p* < 0.05) (Figure 10A). Analysis of tumors after four weeks of the treatment with by liposomal FOXM1 siRNA treatments showed significant FOXM1 protein levels down modulation compared to control tumors (Figure 10B).

## DISCUSSION

FOXM1 transcription factor is considered as one of the key transcriptional drivers of TNBC and the master regulator of tumor metastasis, promoting cell invasion and disease progression. [6, 7]. However, downstream molecular targets of FOXM1 still remains to be determined. eEF2K is an emerging therapeutic target in TNBC and other aggressive cancers including pancreatic, and colon cancers and glioblastoma, and its overexpression correlates with poor patient survival [36–40, 45]. However, the molecular mechanisms regulating eEF2K gene expression is largely unknown. Here we provided the first evidence that eEF2K expression is transcriptionally regulated by FOXM1 and that the FOXM1/eEF2K axis promotes cell TNBC proliferation, survival, migration/invasion and contributes to tumor growth and progression.

The FOXM1-B and FOXM1-C isoforms have been shown to be elevated in numerous human cancers [7]. Several studies demonstrated that FOXM1-B enhances cell migration and invasion, while FOXM1-C promotes cell proliferation, cell migration and invasion in cervical and ovarian cancers [24, 41]. Both FOXM1-B and FOXM1-C play important roles in pancreatic cancer cell biology, but FOXM1-C is more relevant to pancreatic cancer development [46]. In our study FOXM1-B and -1-C had similar effects on eEF2K expression and cell proliferation, migration, invasion and *in vivo* tumor growth in TNBC model.

Constitutive activation of ERK signaling is associated with neoplastic transformation and plays crucial roles in cell survival, apoptosis, and migration during TNBC development and progression [41, 47, 48]. Our results indicate that FOXM1 regulates ERK signaling in breast cancer cells and support previous findings suggesting that FOXM1 is one of the major upstream effectors of ERK signaling in human hepatocellular carcinoma [49]. ERK activation has also been shown to be involved in FOXM1 phosphorylation, nuclear translocation, and enhanced FOXM1 transcriptional activity and to promote downstream target gene expression in some cancers [41, 50]. ERK activity is increased in breast cancer and associated with reduced cell differentiation, larger tumors, higher disease stages, and poorer prognosis in breast cancer [51]. Interestingly, in our study the activity of ERK was not altered by FOXM1 inhibition in MDA-MB-231 cells, which have K-Ras and p53 mutations, suggesting that constitutive K-Ras activity may override FOXM1-mediated ERK activation [52].

FOXM1 is known to regulate the expression of a number of cell cycle genes, including the cyclin D1 gene [9, 3, 10, 11]. Cyclin D1 plays a key role in G1 phase, G1/S transition, and oncogenesis [53]. In the present study, knockdown of FOXM1 led to a marked reduction in cyclin D1 expression, which was recently shown to be regulated by eEF2K in breast cancer cells [36]. Our observations confirm the earlier studies indicating that the FOXM1/eEF2K axis plays a role in cell cycle progression. Considering its role in promotion cell cycle entrance, significant down regulation (~90% reduction) of CyclinD1 in all cell lines following with FOXM1 siRNA treatments, cell cycle inhibition may be the major cause of almost complete abrogation of the colony formation rather than apoptosis induction. Furthermore, the oncogenic effects of cyclin D1 is well established and inhibition of CyclinD1 inhibits not only tumor cell growth or proliferation also suppresses tumor growth in mice, indicating the significance of FOXM1/EF2K/CyclinD1 axis in TNBC cancer biology and progression.

Overall, FOXM1 promotes important biological processes in TNBC cells, including cell survival, proliferation, invasion, migration, and tumorigenesis through regulating multiple signaling pathways including eEF2K and inhibition of FOXM1/eEF2K axis significantly block these events and tumor growth of TNBC, indicating that FOXM1 is a critical driver of progression of breast cancer and other cancers, representing a potential therapeutic target [5, 9, 10, 11]. Our results indicate that the FOXM1 plays an important role in breast cancer progression by transcriptionally regulating eEF2-Kinase expression which is an equally important and an emerging target in TNBC and other solid tumors.

## MATERIALS AND METHODS

### Cell lines, culture conditions, and reagents

Human breast cancer cell lines were obtained from the American Type Culture Collection (Manassas, VA). All cell lines were cultured in Dulbecco modified Eagle medium/F12 supplemented with 10% fetal bovine serum (Sigma-Aldrich, St. Louis, MO), except for non-tumorigenic human breast cells (MCF10A from ATCC), which were cultured in Dulbecco modified Eagle medium/F12 supplemented with 5% horse serum, epidermal growth factor, hydrocortisone, insulin, and cholera toxin (Calbiochem). Cells were cultured at 37°C in a humidified incubator with 5% CO_2_.

### Transfection with siRNA

Two different FOXM1 small interfering RNAs (siRNAs) and non-silencing control siRNAs were purchased from Sigma-Aldrich. Exponentially growing untreated cells were plated 24 h before transfection. Plated cells were transfected with FOXM1 siRNA or a control siRNA at a final concentration of 50 nM for 72 h, using HiPerFect Transfection Reagent (Qiagen, Valencia, CA) according to the manufacturer's protocol. The concentrations of siRNAs were chosen based on dose-response studies. Non-silencing control siRNA-transfected cells were used as negative controls. After treatment, the cells were harvested and processed for further analysis.

### Transfection of MDA-MB-231 cells with a FOXM1 expression vector

A FOXM1 expression plasmid was purchased from Sino Biological Inc. (Beijing, China; catalog number HG12392-G-N). MDA-MB-231 cells were transfected with the FOXM1 expression vector or an empty control vector (400 ng/well) using HiPerFect. At 40 h after transfection, the cells were collected and protein levels were analyzed by Western blotting.

### Western blot analysis

Cells were seeded in 25-cm^2^ culture flasks (3.5 × 10^5^ cells/4 ml medium). Following treatment, the cells were collected, washed twice in ice-cold phosphate-buffered saline (PBS) and lysed in a lysis buffer at 4°C. The total protein concentration for each sample was determined with a detergent-compatible protein assay kit (DC kit; Bio-Rad, Hercules, CA), and Western blotting was performed. Aliquots containing 40 μg of total protein from each sample were analyzed by sodium dodecyl sulfate (SDS)-polyacrylamide gel electrophoresis with a 4%-to-20% gradient for protein separation and electrotransferred to polyvinylidene difluoride membranes. The membranes were blocked with a blocking buffer (0.1 Triton X-100 with 5% dry milk in Tris-buffered saline–Tween 20 [TBS-T]) for 60 min. After being washed with TBS-T, the membranes were probed with the following primary antibodies: FOXM1 (C-20, Santa Cruz Biotechnology, Dallas, TX), extracellular signal-regulated kinase (ERK), and p-ERK (Thr-202) (Santa Cruz Biotechnology, Dallas, TX) and eEF-2K, p-EF2 (Thr-56), EF2, cyclin D1, Src, and p-Src (Tyr-416) (Cell Signaling Technology, Danvers, MA). After being washed with TBS-T, the membranes were incubated with horseradish peroxidase-conjugated anti-rabbit or anti-mouse secondary antibody (Amersham Life Science, Cleveland, OH). Mouse anti-β-actin (primary) and donkey anti-mouse (secondary) (Sigma Chemical, St, Louis, MO) was used as a loading control. All antibodies were diluted in TBS-T containing 5% dry milk. Chemiluminescence detection was performed with ChemiGlow detection reagents (ProteinSimple; Labtech, Uckfield, United Kingdom), and the blots were visualized with a FluorChem 8900 Imager and quantified with a densitometer using the imager application program (Alpha Innotech, San Leandro, CA).

### Cell viability and proliferation assays

Cell viability and proliferation were measured by MTS (3-(4, 5-dimethylthiazol-2-yl)-5-(3-carboxymethoxyphenyl)-2-(4-sulfophenyl)-2H-tetrazolium) assay (Promega, Madison, WI) after treatment. Cells were counted using a hemocytometer, and viable cells were identified by trypan blue exclusion. The identified cells were seeded in 96-well plates (1.25 × 10^3^ cells/well) and transfected with siRNAs. After 72 h of incubation, a solution containing MTS and phenazine methosulfate (20:1 v/v) was added to the cells. After 2–3 h of incubation at 37°C, the number of viable growing cells was estimated by measuring absorption at 490 nm, based on generation of formazan by the cells.

### RNA isolation and reverse transcriptase-PCR (RT-PCR)

Following treatment, total cellular RNA was isolated from the collected cells with TRIzol Reagent (Life Technologies, Carlsbad, CA), and complementary DNA (cDNA) was obtained from 1 μg of total RNA using the RevertAid First Strand cDNA Synthesis Kit (Life Technologies) The cDNAs for FOXM1B, FOXM1C, eEF2K and GAPDH were amplified using the Platinum Taq DNA Polymerase kit (Life Technologies) with specific gene primers. Briefly, 2 μl of the total 20 μl of the reverse-transcribed product was used for PCR in 1x PCR buffer containing 1.5 mM MgCl_2_; 200 μM deoxynucleotide triphosphates; 1 unit of Platinum Taq polymerase; and 0.2 μM FOXM1B-, FOXM1C-, or eEF2K- (Integrated DNA Technologies, Coralville, IA) or GAPDH (Life Technologies)-specific primer. Primer sequences were as follows:

FOXM1B sense, 5′-TTGCCCCCAAGGTGCTGC TA-3′ (41)

FOXM1B antisense, 5′- GGAGATTGGGACGAATC CTC-3′ (41)

*FOXM1C* sense, 5′-CACCCATCACCAGCTTGT TT-3′ (41)

FOXM1C antisense, 5′-GGAGATTGGGACGAATCC TC-3′ (41)

eEF2K sense, 5′-GGAGAGAGTCGAAGGTC ACG-3′ (37)

eEF2K antisense, 5′-GCAATCAGCCAAGACCAT CT-3′’ (37)

The cDNA samples were incubated at 94°C (2–5 min) to denature the template and activate the enzyme. This step was followed by 35 cycles of PCR amplification (as 94°C for 5 min, 95°C for 30 sec, 57°C for 30 sec, and 72°C for 30 sec with FOXM1B and FOXM1C primers; 94°C for 30 sec, 55°C for 30 sec, and 72°C for 60 sec with eEF2K primers, in each cycle). The PCR reaction was terminated with final extension step of 10 min and 5 min at 72°C, respectively. The amplified reaction products were analyzed on a 1.2% agarose gel containing ethidium bromide. The relative amounts of gene products were verified by detection of the GAPDH transcript, which was used as an internal control.

### Colony formation and clonogenic assays

MDA-MB-231 and BT-20 cells were seeded in 6-well plates (1.5 × 10^3^ cells/well); transfected with a non- silencing control siRNA or two different FOXM1siRNAs (50 nM), and grown for 2 weeks. The cells were washed with PBS and stained with crystal violet, and visible colonies were counted.

### Matrigel invasion assay

MDA-MB-231 cells were transfected with 50 nM siRNA, and 72 h later, equal numbers of treated viable cells (5 × 10^4^ cells) were seeded onto Matrigel-coated Transwell filters (8-μm-pore-size) in Matrigel Invasion Chambers (BD Biosciences, San Jose, CA). The number of cells that invaded the lower side of the membrane after 24 h was determined by counting cells in a minimum of four randomly selected areas.

### Cell migration and motility

Cells were seeded in six-well plates (5 × 10^5^ cells/well) and 24 h later were transfected with the control siRNA or two different FOXM1 siRNAs (50 nM). After 72-h incubation, a scratch was created in the monolayer in each well using the tip of a sterile 1000-μl pipette, the cells were gently washed with medium to remove detached cells, and fresh medium was added. Cells in the scratched area were imaged at 0 and 48 h using microscopy, and the distance traveled by cells at the leading edge of the wound at each time point was measured. The results were expressed as percent migration.

### Analysis of apoptosis

Apoptosis was assessed by annexin V staining and flow cytometry analysis. Cells were seeded in 25-cm^2^ culture flasks (3.5 × 10^5^ cells/flask), transfected with siRNA (50 nM) for 72 h, and then analyzed by annexin V/propidium iodide staining according to the manufacturer's protocol (FITC–Annexin V kit; BD Pharmingen, San Diego, CA). Positive cells were detected and quantified by fluorescence-activated cell sorting analysis (FACS).

### Chromatin immunoprecipitation (ChIP) assay

To assess the direct binding of FOXM1 transcription factor to the promoter region of the eEF2K DNA sequence, a chromatin immunoprecipitation (ChIP) assay was performed. Briefly, MDA-MB-231 cells were cross-linked *in situ* by addition of 37% formaldehyde (Fisher Scientific, Pittsburgh, PA) to a final concentration of 1% and incubated at room temperature for 10 min with gentle swirling. The cross-linking reaction was stopped by addition of 2.5 M glycine to a final concentration of 0.125 M, and the cells were incubated at room temperature for 5 min with gentle swirling. The medium was aspirated, and the cells were washed twice with cold PBS and then collected by adding 2 ml of cold PBS containing protease inhibitors (EZ-ChIP kit 17-371; Millipore, Billerica, MA). Cells were scraped from the dish and transferred into an Eppendorf tube, which was centrifuged at 700 × g at 4°C for 5 minutes to pellet cells. The cell pellets were then resuspended in 1 ml of SDS lysis buffer containing protease inhibitors (EZ-ChIP 17-371; 1 ml of SDS lysis buffer for every 1 × 10^7^ MDA-MB-231 cells) and placed on ice. The resulting extract was sonicated to create DNA fragments between 200 and 1000 bp using a ultrasonic sonicator (Sonicator, Heat Systems-Ultrasonics, INC, model W-225). The extract was incubated on ice. At this stage, the processing of all experimental samples and total input was carried out according to the Milli pore ChIP assay protocol (EZ-ChIP 17-371). Ten microliters (1%) of each sample was used as input. The remaining samples were used for immunoprecipitation with 10 μl of anti-FOXM1 antibody (H-300; Santa Cruz Biotechnology), 1 μg of a rabbit antibody (negative control; Amersham Life Science, Cleveland, OH), 1 μg of anti-RNA polymerase II (positive control; EZ-ChIP 05-623B; Millipore), or 1 μg of normal mouse immunoglobulin G (IgG) serum (negative control) (EZ-ChIP 12-371B; Millipore). After the addition of an antibody, each sample was incubated overnight at 4°C with rotation and washed according to the Upstate Biotechnology ChIP assay protocol. Cross-links were reversed for all samples, including input, by addition of 8 μl of 5 M NaCl, and then the samples were incubated overnight at 65°C. One microliter of RNase was then added, and the samples were incubated for 30 min at 37°C. Proteinase K (1 μl), Tris-HCl (1 M, 8 μl), and EDTA (0.5 M, 4 μl) were added, and the samples were digested for 2 h at 45°C. DNA was extracted from the digested samples using PCR purification columns (EZ-ChIP 20-290 and 20-291; Millipore). Two microliters of each extracted DNA sample (the input sample and ChIP DNA sample) was used for PCR amplification in 35 cycles using primers specific to promoter fragments of the eEF2K gene and control primers.

### PCR primers and reaction conditions for ChIP assay

Control primers (EZ-ChIP 22-004; Millipore) were used for the human GAPDH gene as an internal control. Two eEF2K promoter regions were identified (with data from the University of California, Santa Cruz, Genome Bioinformatics website, http://genome.ucsc.edu), Ten different primers were designed for two promoter regions of the eEF2K gene using the Primer3 program (SimGene.com). The following primers were used to amplify the EF2K gene promoter (with position numbers relative to the transcription start site):

BS1 sense, position −145, 5′-CAAGCTATCCT CCCACCTCA-3′

BS1 antisense, position 341, 5′-TGGCTCATGCCTG TAATCCT-3′

BS2 sense, position −1358, 5′ATCTGCTCAACT CCCTGGAA-3′

BS2 sense, position −1358, 5′ATCTGCTCAACTC CCTGGAA-3′

BS2 antisense, position −1558, 5′-GAAAATAGC CCTCCCCACTC-3′

BS3 sense, position −1361, 5′-TGCTCAACTCC CTGGAAAAG-3′

BS3 antisense, position −1558, 5′- GAAAATAGCC CTCCCCACTC -3′

BS4 sense, position −2256, 5′-AGTGCTGGGAAGA TGGAATG-3′

BS4 antisense, position −2454, 5′- CCTGTGGCAT GAGTGGTAGA-3′

BS5 sense, position −2259, 5′-GCTGGGAAG ATGGAATGAGA-3′

BS5 antisense, position −2454, 5′-CCTGTGGCA TGAGTGGTAGA-3′

BS6 sense, position −144, 5′- CAAGCTATCCT CCCACCTCA-3′

BS6 antisense, position −341, 5′-TGGCTCA TGCCTGTAATCCT-3′

BS7 sense, position −1359, 5′- CTGCTCAACTCCC TGGAAAA-3′

BS7 antisense, position −1558, 5′- GAAAATAGCC CTCCCCACTC-3′

BS8 sense, position −1360, 5′- AGCACCATATTT GGCACACA-3′

BS8 antisense, position −1558, 5′- GCCTCATTGAT TGGTTCAGG-3′

BS9 sense, position −2257, 5′- AGTGCTGGGAA GATGGAATG-3′

BS9 antisense, position −2454, 5′- CCTGTGGCATG AGTGGTAGA-3′

BS10 sense, position −2256, 5′- AGTGCTGGGAA GATGGAATG-3′

BS10 antisense, position −2450, 5′- TGGCATGAGT GGTAGAGTGG-3′

Specific primers were designed for the promoter region (−1558/−1359) of the eEF2K gene where FOXM1 bindinga sides are located. The region was amplified using the following primers: 5′- TGGTTACTATAA AAGGCCCAGAT -3′ (sense) and 5′- CAGACCTGGCCA ATTAGCAT -3′ (antisense). The following the reaction, the mixture was used for all PCR samples: 2 μl of each purified ChIP extract, input for PCR in 1x PCR buffer containing 1.5 mM MgCl_2_, 200 μM deoxynucleotide triphosphates, 1 unit of Platinum Taq polymerase, and 0.2 μM each primer (Integrated DNA Technologies) in a 25-μl total volume. The DNA samples were incubated at 94°C (3 min). This step was followed by 35 cycles of PCR (94°C for 30 sec, 58°C for 30 sec, and 72°C for 1 min). The amplified reaction products were analyzed on a 1.2% agarose gel containing ethidium bromide.

### Plasmid constructs and luciferase reporter assay

Fragments containing the predicted binding sites (−1558/−1359 region in the promoter of the human eEF2K gene) were amplified genomic DNA extracted from MDA-MB-231 cells by PCR using the following primers: 5′-agcaagcttctgctcaactccctggaaaa-3′ (sense) and 5′-AGCAAGCTTgaaaatagccctccccactc-3′ (antisense) (Integrated DNA Technologies). The PCR-amplified promoter regions were digested with HindIII (New Englands Biolabs, Ipswich, MA) and inserted into a pGL3-Basic empty vector (Promega) using T4 DNA ligase (New England Biolabs). The plasmid minipreparations were performed with the QIAprep Spin Miniprep kit (Qiagen). The eEF2K promoter region was confirmed by DNA sequencing (Lone Star Labs Inc., Houston, TX). In addition to, pGL3 containing the eEF2K DNA promoter region, pGL3-Basic empty vector, a FOXM1 expression vector (Sino Biological Inc.), and the renilla luciferase expression plasmid pRLTK was used as internal controls (Promega).

To up-regulate FOXM1 expression, a pCMV-FOXM1 expression vector was transfected into MDA-MB-231 cells using HiPerFect. To silence FOXM1 expression, MDA-MB-231 cells were seeded in 24-well plates (8 × 10^4^ cells/well) and transfected with FOXM1 siRNA or control siRNA. After 24-h incubation, the cells were transfected with non-silencing control siRNA or FOXM1 siRNA (50 nM) and 2 ng/μl of pCMV-FOXM1 expression vector. Twenty-four hours after transfection, cells were co-transfected with 2 ng/μl of the pGL3-Basic vector, 2 ng/μl of pGL3 containing the eEF2K DNA promoter region, 2 ng/μl of the pGL3 control expression vector, and 0.8 ng/μl of the pRLTK renilla luciferase expression plasmid. Plasmids and siRNAs were transfected into MDA-MB-231 cells using HiPerFect. At 40 h after transfection, cells were harvested and protein extracts were prepared for the Dual-Luciferase Reporter Assay (Promega). Luciferase activity in control or FOXM1siRNA-transfected cells was normalized for transfection efficiency to renilla luciferase activity, and the results were compared with those for the cells transfected with the pGL3-Basic empty vector.

### Preparation of liposomal siRNA nanoparticles

*In vivo* siRNA delivery was achieved by incorporating siRNA into liposomes consisted of *Dimyristoyl*-*sn*-*glycero*-*3*-*phosphocholine* (DMPC) and pegylated distreroly-phosphotidyl ethanolamine (DSPE-PEG-2000) (Avanti Lipids). DMPC and DSPE-PEG2000 were mixed at the ratio of (10:1) and siRNA were mixed at a ratio of 10:1 lipid to oligos (control siRNAor FOXM1 siRNA) (w/w) in the presence of excess tertiary butanol. Prior to *in vivo* administration, the siRNA/lipid complex was reconstituted in saline and systemically administered (0.3 mg/kg or 8 μg/mouse) once a week in a volume of 100 μl.

### *In vivo* tumor xenograft model of TN breast cancer

Athymic Nu/Nu female mice (4–5 week old) were obtained from the Department of Experimental Radiation Oncology at M. D. Anderson Cancer Center, Houston, TX. All studies were conducted according to an experimental protocol approved by the M. D. Anderson Institutional Animal Care and Use Committee. MDA-MB-231 cells (2 × 10^6^ in 20% matrigel) were injected into the mammary fat pad of each mouse. Two weeks after injection, when tumors reached about 3–5 mm in size, liposomal siRNA treatments (I.v from tail vein) were initiated. Each mouse received 0.3 mg/kg siRNA (control or FOXM1 siRNA) incorporated in liposomes once a week (equivalent of 8 ug/mouse) for four weeks and tumor volumes were measured weakly by a caliper. After completion of treatments, mice were euthanized with CO_2_, tumor tissues were removed, lysed and analyzed by Western blot.

### Statistical analysis

All experiments were conducted at least in triplicate, and the results were summarized as means with standard deviations. Statistical significance was determined using the Student *t* test. *P*-values less than 0.05 were considered statistically significant.

## SUPPLEMENTARY MATERIALS FIGURES


