# Supplementary material for: FOXM1 regulates expression of eukaryotic elongation factor 2 kinase and promotes proliferation, invasion and tumorgenesis of human triple negative breast cancer cells
Source: Oncotarget. 2016 Feb 24;7(13):16619–35. doi: 10.18632/oncotarget.7672 (PMC4941339; doi:10.18632/oncotarget.7672)
Supplement: Supplementary file 1 [file oncotarget-07-16619-s001.pdf]

FOXM1 regulates expression of eukaryotic elongation factor 2 kinase and promotes proliferation, invasion and tumorgenesis of human triple negative breast cancer cells

Supplementary Materials

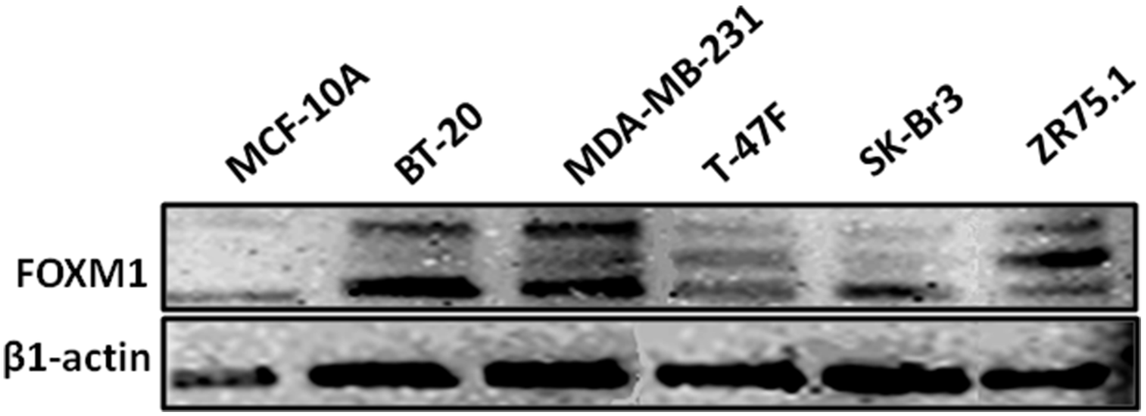

Supplementary Figure S1: The expression levels of FOXM1 were lower in the ER-positive T-47D and ZR-75.1 cells and HER2/Neu-overexpressing SKBR3 cells than in TNBC cells.

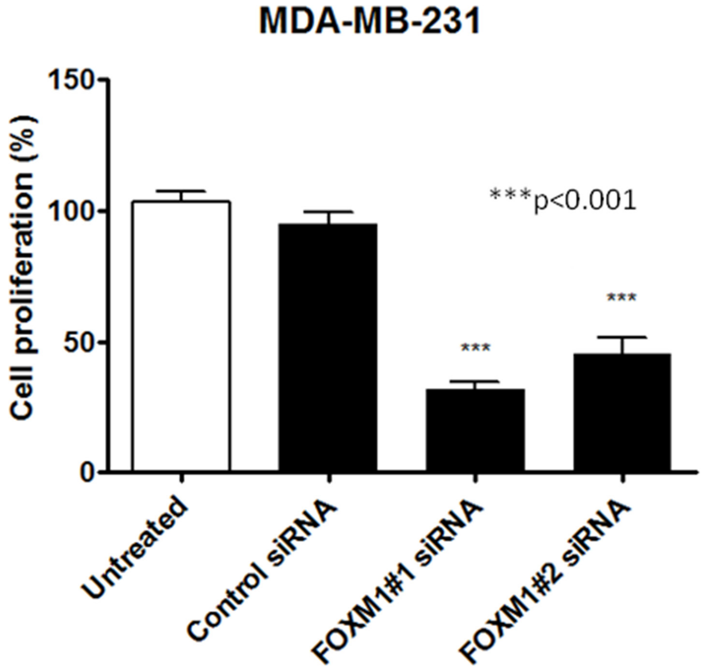

Supplementary Figure S2: MDA-MB-231 cells were transfected with FOXM1#1 or FOXM1#2 siRNA, and after 72 h, cell proliferation was measured by an MTS assay. Knockdown of FOXM1 significantly inhibited cell proliferation. The data are means with standard deviations. *represents significant difference between indicated groups.

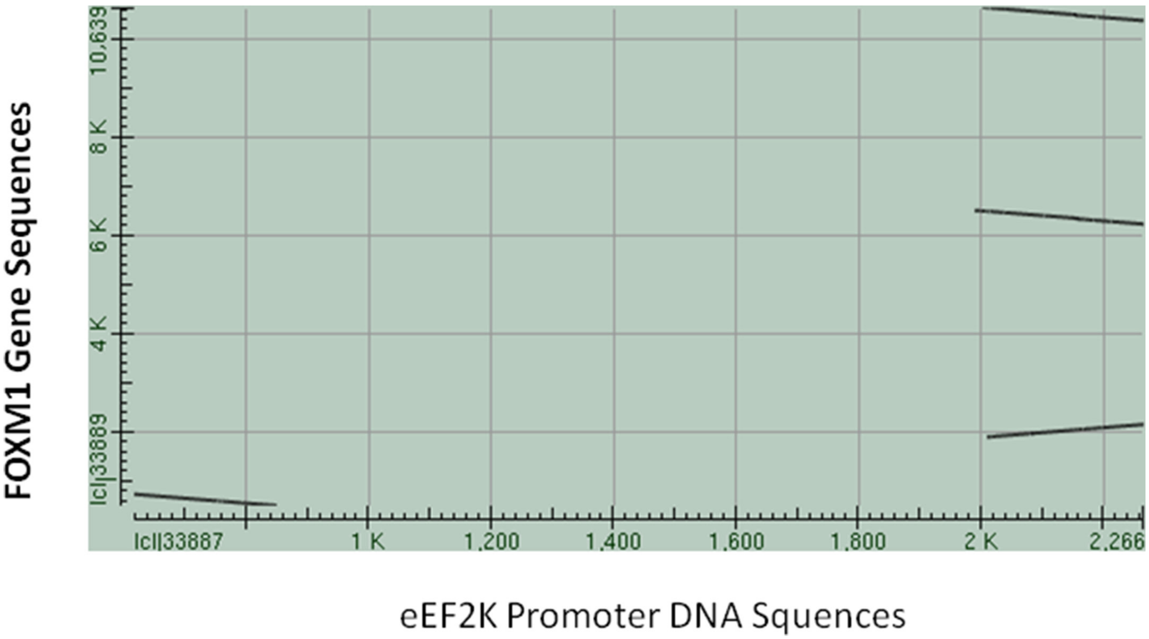

Supplementary Figure S3: Schematic representation of consensus between eEF2K promoter sequences and FOXM1 gene sequences according to the Align Sequences Nucleotide BLAST program. There were four different FOXM1-binding consensus sequences in the eEF2K promoter region.
